# Effects of Dietary Sweet Potato Tuber Meal on Production Performance, Meat Quality and Intestine of Wenchang Chickens

**DOI:** 10.3390/biology15120955

**Published:** 2026-06-18

**Authors:** Jingli Yuan, Jie Liu, Limin Wei, Qiqi Guo, Yan Zhang, Xiuping Wang, Guiping Zhao, Quanwei Liu

**Affiliations:** 1 Xia Xianzhu Academician Team Innovation Center, Hainan Key Laboratory of Tropical Animal Breeding and Epidemic Research, Institute of Animal Husbandry & Veterinary Research, Hainan Academy of Agricultural Sciences, Haikou 571100, China; 13250732023@163.com (J.Y.);; 2 Sanya Institute, Hainan Academy of Agricultural Sciences (Hainan Experimental Animal Research Center), Sanya 572025, China; 3 Key Laboratory of Wenchang Chicken Breeding and Feeding, Hainan (Tan Niu) Wenchang Chicken Co., Ltd., Haikou 571100, China

**Keywords:** Wenchang chicken, sweet potato tuber meal, physiological parameters, intestinal

## Abstract

This study was conducted to investigate the effects of dietary supplementation with graded levels of SPTM on growth performance, slaughter performance, physiological parameters, and jejunal morphology of Wenchang chickens. A total of 400 female Wenchang chickens at 81 days of age with the same genetic background and similar body weight (1190.80 ± 5.54 g) were randomly allocated into four treatment groups with five replicates per group and 20 chickens per replicate. Birds were fed diets supplemented with 0%, 3%, 9%, and 12% SPTM, respectively. The experimental period lasted 40 days. The results showed that dietary SPTM supplementation had no significant effects on growth performance, slaughter performance, organ indices, or serum biochemical parameters (*p* > 0.05). However, significant effects were observed on serum enzyme activities, immune parameters, jejunal morphology, meat quality, and nutrient composition. Specifically, serum alanine aminotransferase (ALT) activity in the 9% SPTM group was significantly lower than that in all other groups (*p* < 0.05). Aspartate aminotransferase (AST) activity in the 3% SPTM group was significantly lower than that in the 12% SPTM group (*p* < 0.05). Breast muscle moisture content in the 12% SPTM group was significantly lower than that in the control group (*p* < 0.05). Total amino acid and threonine contents in the breast muscle of the 12% SPTM group were significantly lower than those in the 0% and 3% SPTM groups (*p* < 0.05). Lauric acid (C12:0) and myristic acid (C14:0) contents in the breast muscle of the 9% and 12% SPTM groups were significantly higher than those in the 0%, and 3% SPTM groups (*p* < 0.05). These selective effects on meat quality traits suggest that SPTM has potential as a partial corn replacer, but further studies are needed to optimize inclusion levels and validate sensory outcomes. This systematic investigation of the effects of SPTM on physiological parameters and meat quality in Wenchang chickens provides a theoretical basis for the rational and efficient utilization of SPTM in Wenchang chicken production.

## Simple Summary

Sweet potato tuber meal (SPTM) is a potential alternative to conventional energy feed ingredients such as corn in poultry diets due to its high starch content and wide availability. However, the optimal inclusion level of SPTM in the diet of indigenous chicken breeds, particularly during the finishing period, remains unclear. This study evaluated the effects of supplementing diets with 0%, 3%, 9%, and 12% SPTM on the growth, slaughter performance, physiological parameters, and jejunal morphology of 81-day-old female Wenchang chickens over a 40-day period. The results showed that dietary SPTM did not negatively affect growth performance, slaughter performance, organ indices, or serum biochemical parameters. Notably, dietary inclusion of 12% SPTM selectively increased certain flavor-related compounds (e.g., IMP) and improved muscle fiber characteristics. These selective effects on meat quality traits suggest that SPTM has potential as a partial corn replacer, but further studies are needed to optimize inclusion levels and validate sensory outcomes.

## 1. Introduction

The continuous expansion of the global intensive poultry industry has driven a surging demand for conventional feed ingredients, predominantly corn and soybean meal [1,2]. Consequently, the escalating costs and the growing competition between human food and animal feed supplies have become primary constraints on the sustainable development of the poultry sector. To mitigate these challenges, exploring and evaluating unconventional, cost-effective, and widely available alternative feed resources has emerged as a critical focus in current animal nutrition research [3].

Sweet potato (*Ipomoea batata* L.), also known as tuberous morning glory, is an important food crop cultivated in over 100 countries worldwide [4]. China ranks first in global sweet potato production annually [5]. The utilization of sweet potato and its processing by-products as non-grain feed resources is of great significance for alleviating the human–livestock competition for grain and reducing feed production costs. The sweet potato tuber is rich in starch (approximately 50–80%), dietary fiber, and various vitamins and minerals, with a gross energy metabolizability of up to 87.88% [6,7]. However, its widespread application is limited by a relatively low crude protein content, an imbalance in essential amino acids, and the presence of minor anti-nutritional factors such as trypsin inhibitors [8]. Previous studies have demonstrated that the moderate substitution of corn with sweet potato or cassava meal in broiler diets does not compromise overall growth performance or slaughter yield [9]. Nonetheless, excessive inclusion levels may burden the gastrointestinal tract, alter intestinal mucosal morphology (e.g., jejunal villus length), and elevate hepatic metabolic load, as reflected by fluctuations in serum ALT and AST activities [10]. Currently, most nutritional evaluations of sweet potato meal have been conducted on fast-growing commercial broilers, leaving a significant gap in understanding its systemic physiological effects on slow-growing, premium indigenous poultry breeds.

Wenchang chicken are a renowned indigenous Chinese yellow-feathered breed, highly valued by consumers for its thin skin, tender texture, and distinctive flavor profile [11]. Unlike fast-growing white-feathered broilers, Wenchang chickens possess a prolonged growth cycle and a unique physiological capacity for intramuscular fat (IMF) and flavor precursor deposition [12]. The late fattening phase (e.g., 81 to 120 days of age) is a critical window for the development of meat quality traits, including the spatial arrangement of muscle fibers, the synthesis of taste-active amino acids, and the accumulation of specific fatty acids (such as lauric acid and myristic acid) [13,14,15]. Research indicates that the microstructural and biochemical properties of meat in slow-growing chickens are highly sensitive to dietary carbohydrate sources and nutritional interventions [16]. Therefore, Wenchang chickens serve as an excellent in vivo model to investigate how unconventional energy feeds modulate meat quality and physiological homeostasis.

Despite the known potential of sweet potato meal, its optimal inclusion level and its specific modulatory effects on the physiological barriers and histological meat characteristics of Wenchang chickens during the late growth phase remain poorly understood. Therefore, the present study aimed to systematically evaluate the effects of dietary sweet potato meal inclusion at varying levels (0%, 3%, 9%, and 12%) on the growth performance, slaughter traits, serum biochemical and immune indices, intestinal morphology, and meat quality (including muscle fiber characteristics, amino acid content, and fatty acid profiles) of Wenchang chickens from 81 to 120 days of age. The findings of this research will provide a robust theoretical basis and practical guidelines for the rational and efficient utilization of sweet potato meal in the production of high-quality poultry.

## 2. Materials and Methods

### 2.1. Experimental Materials and Animals

SPTM was purchased from a wholesale market in Wenchang City, Hainan Province, China. The sweet potato tubers were washed, sliced, sun-dried for 48 h, and then ground to pass through a 0.5-mm sieve. No heat treatment was applied, which may preserve some trypsin inhibitor activity. The nutrient composition of the SPTM was as follows: dry matter, 88.90%; crude protein, 4.36%; crude fat, 1.50%; crude ash, 3.70%; calcium, 0.22%; total phosphorus, 0.17%; and crude fiber, 3.90%. The experimental chickens were obtained from Hainan (Tanniu) Wenchang Chicken Co., Ltd. (Haikou, China).

### 2.2. Experimental Design

A total of 400 healthy 81-day-old female Wenchang chickens with uniform genetic background and similar body weight were randomly allocated into four treatment groups, each consisting of five replicates with 20 chickens per replicate (*n* = 10 per treatment, 2 per pen). The chickens were fed a basal diet supplemented with 0%, 3%, 9%, and 12% SPTM, respectively. The experimental diets were formulated according to the Nutrient Requirements of Yellow-Feathered Broilers (Ministry of Agriculture of the People’s Republic of China, 2020 revision). The ingredient composition and nutrient content of the experimental diets are presented in Table 1. The 40-day feeding trial was conducted at the Yongfa Research Base of the Institute of Animal Science and Veterinary Medicine, Hainan Academy of Agricultural Sciences. The chickens were reared under a combination of artificial and natural lighting, with ad libitum access to feed and water. Routine vaccination programs and standard management practices were followed. The experimental design and procedures were approved by the Institutional Animal Care and Use Committee of the Institute of Animal Science and Veterinary Medicine, Hainan Academy of Agricultural Sciences (Approval No.: HNSYY20230721).

### 2.3. Parameter Measurement and Methods

#### 2.3.1. Growth Performance Measurement

At the beginning and end of the experiment, the chickens were individually weighed. Daily feed intake and residual feed were recorded throughout the experimental period. Average daily gain (ADG), average daily feed intake (ADFI), and feed-to-gain ratios (F/G) were calculated.

#### 2.3.2. Slaughter Performance Measurement

At the end of the experiment, two chickens from each replicate with body weights close to the replicate average were selected, a total of 40 chickens were slaughtered across the four groups for slaughter performance determination according to the standard methods described in Terms and Statistical Methods for Growth Performance of Poultry (NY/T 823-2004). The following parameters were measured: live weight, carcass weight, semi-eviscerated weight, eviscerated weight, leg muscle weight, breast muscle weight, and abdominal fat weight. The following indices were subsequently calculated: dressing percentage, semi-eviscerated percentage, eviscerated percentage, leg muscle percentage, breast muscle percentage, and abdominal fat percentage.

#### 2.3.3. Organ Index Measurement

After slaughter, the stomach (including proventriculus and gizzard), heart, liver, and spleen were collected. Excess moisture and blood were removed using filter paper, and adhering tissues and fat were carefully trimmed before weighing. The organ index was calculated as follows:

Organ index (g/kg) = Organ weight (g)/Live weight (kg)

#### 2.3.4. Serum Biochemical, Antioxidant, and Immune Index Determination

Two chickens from each replicate of different treatment groups were selected for blood sampling. Approximately 5 mL of blood was collected from each bird via the anterior vena cava. Blood samples were centrifuged at 4000 r/min for 15 min at 4 °C to separate serum. The serum was then stored at −20 °C for subsequent analysis of serum biochemical, antioxidant, and immune parameters.

Commercially available assay kits (purchased from Nanjing Jiancheng Technology Co., Ltd., Nanjing, China) were used to determine serum levels of total protein (TP), albumin (ALB), glucose (GLU), uric acid (UA), total cholesterol (TC), triglycerides (TGs), alkaline phosphatase (ALP), AST, and ALT as described in Liu et al. [17]. Globulin (GLB) levels were calculated by subtracting ALB from TP.

#### 2.3.5. Intestinal Morphology

At the end of the experiment, two chickens from each replicate were randomly selected. After slaughter, approximately 1 cm of the proximal jejunum was rapidly excised, rinsed with physiological saline, and fixed in 4% paraformaldehyde solution for at least 24 h for subsequent histological analysis. The fixed jejunal segments were then dehydrated, cleared, embedded in paraffin, sectioned (at approximately 5 μm thickness), and stained with hematoxylin and eosin (H&E). Villus height and crypt depth were measured using a light microscope. The villus height-to-crypt depth ratio (V/C) was subsequently calculated.

#### 2.3.6. Meat Quality and Nutrient Content Determination

The pH value, meat color, drip loss, cooking loss, and shear force of the pectoralis major (breast muscle) and biceps femoris (leg muscle) were determined according to established procedures used in poultry meat quality studies. Briefly, muscle pH (Testo 205 or equivalent) was measured at pH_45min_ and pH_24h_ using a calibrated portable pH meter. The instrument was calibrated before each measurement using standard buffer solutions at pH 4.00 and 7.00 (NIST-traceable buffer solutions, Mettler Toledo, Columbus, OH, USA), and measurements were performed by inserting the electrode directly into the center of the muscle sample at three different locations, with the mean value recorded. Meat color (L*, a*, b*) was determined at 24 h postmortem on the freshly cut surface of the muscle after blooming for 30 min at 4 °C using a colorimeter (CR-400, Konica Minolta Sensing Inc., Osaka, Japan) with standard calibration using a white tile. Drip loss was measured by suspending standardized muscle samples (approximately 20–30 g) in sealed plastic bags at 4 °C for 24 h and expressed as percentage weight loss relative to the initial weight. Cooking loss was determined by heating vacuum-packed samples in a thermostatically controlled water bath at 80 °C until the core temperature reached 75 °C, followed by cooling to room temperature and calculating percentage weight loss. Shear force was measured on cooked samples using a texture analyzer (TA.XT Plus, Stable Micro Systems Ltd., Surrey, UK) equipped with a Warner-Bratzler blade. Each sample was cut into 1.0 × 1.0 × 3.0 cm strips, and the maximum shear force was recorded. Approximately 200–300 g of breast and leg muscle samples were collected at 24 h postmortem, immediately frozen in liquid nitrogen, and stored at −80 °C for subsequent analysis of dry matter, crude protein, IMF, IMP, fatty acid composition, and amino acid profile. IMF was determined using Soxhlet ether extraction. IMP content was analyzed by high-performance liquid chromatography (HPLC). Fatty acid composition was determined by gas chromatography (GC) after lipid extraction and methyl esterification. Amino acid composition was determined using an automatic amino acid analyzer following acid hydrolysis of muscle protein. All measurements were performed in triplicate or duplicate where applicable, and the mean value was used for statistical analysis.

#### 2.3.7. Muscle Fiber Morphology

At the end of the experiment, two chickens from each replicate were randomly selected. Approximately 20 g of breast and leg muscle samples were collected and fixed in 4% paraformaldehyde solution for 24 h. Tissue sections were prepared and stained with H&E. Muscle fiber diameter, fiber density, and cross-sectional area of breast muscle were observed under a light microscope. Five random fields per section at 400× magnification were analyzed. Histological images were captured using CaseViewer 2.2 software (3DHISTECH, Budapest, Hungary). Morphometric analysis was performed using Image-Pro Plus 6.0 software (Media Cybernetics, Rockville, MD, USA) with manual thresholding. Fiber number, area, and diameter were measured. Analyst was blinded to treatment.

### 2.4. Statistical Analysis

Data were subjected to one-way analysis of variance (ANOVA) using the GLM procedure of SAS 9.2. When significant treatment effects were detected, differences among means were separated using Duncan’s multiple-range test. In addition, orthogonal polynomial contrasts were performed to evaluate the linear and quadratic responses to increasing dietary levels of sweet potato tuber meal (SPTM; 0%, 3%, 9%, and 12%). Mortality data were analyzed as proportional data using the χ^2^ test. Results are presented as means ± SEM. Statistical significance was declared at *p* < 0.05, and 0.05 ≤ *p* < 0.10 was considered a tendency. Considering the large number of response variables evaluated, FDR adjustment using the Benjamini–Hochberg procedure was additionally performed as a sensitivity analysis for key outcomes. The overall interpretation of the results was not materially altered after FDR correction.

## 3. Results

### 3.1. Effects of Different Levels of SPTM on Growth Performance of Wenchang Chickens

The effects of different levels of SPTM on growth performance of Wenchang chickens aged 81–120 days are presented in Table 2. As shown in Table 2, no significant differences (*p* > 0.05) were observed among treatment groups in terms of final body weight, average daily gain, average daily feed intake and feed-to-gain ratio.

### 3.2. Effects of Different Levels of SPTM on Slaughter Performance of Wenchang Chickens

The effects of different levels of SPTM on slaughter performance of Wenchang chickens are presented in Table 3. As shown in Table 3, no significant differences (*p* > 0.05) were observed among groups in dressing percentage, semi-eviscerated percentage, eviscerated percentage, abdominal fat percentage, leg muscle percentage, or breast muscle percentage.

### 3.3. Effects of Different Levels of SPTM on Organ Indices of Wenchang Chickens

The effects of different levels of SPTM on organ indices of Wenchang chickens are presented in Table 4. As shown in Table 4, no significant differences (*p* > 0.05) were observed among groups in any of the organ indices measured.

### 3.4. Effects of Different Levels of SPTM on Serum Biochemical Parameters of Wenchang Chickens

The effects of different levels of SPTM on serum biochemical parameters of Wenchang chickens aged 81–120 days are presented in Table 5. As shown in Table 5, no significant differences (*p* > 0.05) were observed among groups in terms of TP, albumin, globulin, glucose, uric acid, triglycerides, or total cholesterol.

### 3.5. Effects of Different Levels of SPTM on Serum Enzyme Activities and Immune Parameters of Wenchang Chickens

The effects of different levels of SPTM on serum enzyme activities and immune parameters of Wenchang chickens aged 81–120 days are presented in Table 6. As shown in Table 6, serum ALT activity in the 9% group was significantly lower than that in all other groups (*p* < 0.05). Serum AST activity in the 3% group was significantly lower than that in the 12% groups (*p* < 0.05), but no significant difference was observed between the 3% group and the 0% or 9% groups (*p* > 0.05). No significant differences (*p* > 0.05) were detected among groups in alkaline phosphatase (ALP) activity or immunoglobulin A (IgA) and immunoglobulin M (IgM) levels.

### 3.6. Effects of Different Levels of SPTM on Jejunal Morphology of Wenchang Chickens

The effects of different levels of SPTM on jejunal morphology of Wenchang chickens are presented in Table 7. As shown in Table 7, jejunal villus height in the 9% group was significantly lower than that in the control (0%) group and the 12% group (*p* < 0.05), but no significant difference was observed between the 9% group and the 3% group (*p* > 0.05). The number of intestinal glands in the 9% group was significantly lower than that in the control group (*p* < 0.05), but no significant differences were observed between the 9% group and the 3% or 12% group (*p* > 0.05). The number of intestinal glands per unit area in the 9% group was significantly lower than that in the control and 12% groups (*p* < 0.05), but no significant difference was observed between the 9% and 3% groups (*p* > 0.05).

### 3.7. Effects of Different Levels of SPTM on Physicochemical Properties of Breast Muscle in Wenchang Chickens

The effects of different levels of SPTM on physicochemical properties of breast muscle in Wenchang chickens are presented in Table 8. As shown in Table 8, no significant differences (*p* > 0.05) were observed among groups in pH, drip loss, lightness (L*), redness (a*), or yellowness (b*).

### 3.8. Effects of Different Levels of SPTM on Physicochemical Properties of Leg Muscle in Wenchang Chickens

The effects of different levels of SPTM on physicochemical properties of leg muscle in Wenchang chickens are presented in Table 9. As shown in Table 9, no significant differences (*p* > 0.05) were observed among groups in any of the physicochemical properties of leg muscle.

### 3.9. Effects of Different Levels of SPTM on Nutrient Composition of Breast Muscle in Wenchang Chickens

The effects of different levels of SPTM on nutrient composition of breast muscle in Wenchang chickens are presented in Table 10. As shown in Table 10, breast muscle moisture content in the 12% group was significantly lower than that in the control (0%) group and the 9% group (*p* < 0.05), but no significant difference was observed between the 12% group and the 3% group (*p* > 0.05). IMF content in the 12% group was significantly higher than that in the control and 3% groups (*p* < 0.05), but no significant difference was observed between the 12% group and the 9% group (*p* > 0.05). IMP content in the 9% group was significantly higher than that in the control and 3% groups (*p* < 0.05), but no significant difference was observed between the 9% group and the 12% group (*p* > 0.05).

### 3.10. Effects of Different Levels of SPTM on Nutrient Composition of Leg Muscle in Wenchang Chickens

The effects of different levels of SPTM on nutrient composition of leg muscle in Wenchang chickens are presented in Table 11. As shown in Table 11, no significant differences (*p* > 0.05) were observed among groups in any of the conventional nutrient composition parameters of leg muscle.

### 3.11. Effects of Different Levels of SPTM on Amino Acid Composition of Breast Muscle in Wenchang Chickens

The effects of different levels of SPTM on amino acid composition of breast muscle in Wenchang chickens are presented in Table 12. As shown in Table 12, total amino acid (TAA) and threonine (Thr) contents in the breast muscle of the 12% group were significantly lower than those in the control (0%) and 3% groups (*p* < 0.05). Essential amino acid (EAA), umami amino acid (UAA), aspartic acid (Asp), and glutamic acid (Glu) contents in the 12% group were significantly lower than those in all other groups (*p* < 0.05). Serine (Ser) contents in the 9% and 12% groups were significantly lower than those in the 0% and 3% groups (*p* < 0.05). Methionine (Met) content in the 0% group was significantly lower than that in the 9% and 12% groups (*p* < 0.05). Leucine (Leu) content in the control group was significantly lower than that in the 3%, 9%, and 12% groups (*p* < 0.05). Tyrosine (Tyr) content in the control and 12% groups was significantly lower than that in the 3% group (*p* < 0.05). Lysine (Lys) content in the control group was significantly lower than that in the 3% group (*p* < 0.05).

### 3.12. Effects of Different Levels of SPTM on Amino Acid Composition of Leg Muscle in Wenchang Chickens

The effects of different levels of SPTM on amino acid composition of leg muscle in Wenchang chickens are presented in Table 13. As shown in Table 13, total amino acid, serine, and lysine contents in the control group (0%) were significantly lower than those in the 3% group (*p* < 0.05). Essential amino acid, umami amino acid, and threonine contents in the control group were significantly lower than those in the 3% group (*p* < 0.05). Aspartic acid content in the control group was significantly lower than that in the 3% and 12% groups (*p* < 0.05). Glutamic acid content in the control group was significantly lower than that in all other groups (*p* < 0.05). Methionine and phenylalanine contents in the control group were significantly lower than those in the 9% and 12% groups (*p* < 0.05). Leucine content in the control group was significantly lower than that in the 3% and 12% groups (*p* < 0.05). Tyrosine content in the control group was significantly lower than that in the 3% and 9% groups (*p* < 0.05).

### 3.13. Effects of Different Levels of SPTM on Fatty Acid Composition of Breast Muscle in Wenchang Chickens

The effects of different levels of SPTM on fatty acid composition of breast muscle in Wenchang chickens are presented in Table 14. As shown in Table 14, total fatty acids (TFAs), saturated fatty acids (SFAs), monounsaturated fatty acids (MUFAs), polyunsaturated fatty acids (PUFAs), as well as palmitic acid (C16:0), stearic acid (C18:0), cis-9-hexadecenoic acid (C16:1), cis,cis-9,12-octadecadienoic acid (C18:2n-6), cis-11-eicosenoic acid (C20:1), α-linolenic acid (C18:3n-3), and cis,cis-11,14-eicosadienoic acid (C20:2) contents in the breast muscle of the 12% group were significantly higher than those in the 0% and 9% groups (*p* < 0.05). Myristic acid (C14:0) and cis-9-hexadecenoic acid (C16:1) contents in the breast muscle of the 12% group were significantly higher than those in all other groups (*p* < 0.05). Arachidonic acid (C20:4n-6) content in the 0% groups was significantly higher than that in the 12% group (*p* < 0.05). Docosahexaenoic acid (DHA, C22:6n-3) content in the 0%, and 9% groups was also significantly higher than that in the 12% group (*p* < 0.05).

### 3.14. Effects of Different Levels of SPTM on Fatty Acid Composition of Leg Muscle in Wenchang Chickens

The effects of different levels of SPTM on fatty acid composition of leg muscle in Wenchang chickens are presented in Table 15. As shown in Table 15, lauric acid (C12:0) and myristic acid (C14:0) contents in the 9% and 12% groups were significantly higher than those in the 0% and 3% groups (*p* < 0.05), and the 3% group also showed significantly higher values than the 0% group (*p* < 0.05). Arachidonic acid (C20:4n-6) content in the 3% group was significantly higher than that in all other groups (*p* < 0.05).

### 3.15. Effects of Different Levels of SPTM on Breast Muscle Fiber Characteristics of Wenchang Chickens

**Figure 1:**
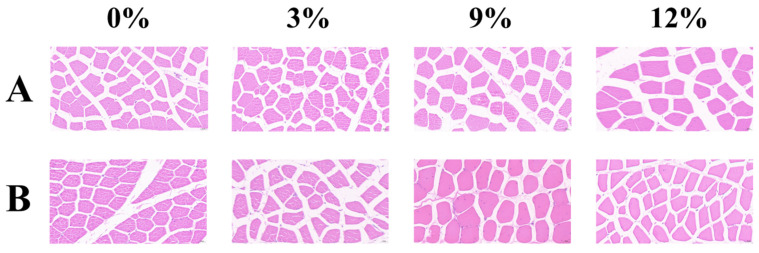
Breast muscle (**A**) and thigh muscle (**B**) histological characteristics, scale bar: 20 µm. Note: Five random fields per section at 400× magnification. Tissue sections were prepared and stained with H&E.

The effects of different levels of SPTM on breast muscle fiber characteristics of Wenchang chickens are presented in Table 16 and Figure 1. As shown in Table 16, the number of breast muscle fibers in the 0% group was significantly higher than that in the 12% group (*p* < 0.05), but no significant differences were observed between the 0% group and the other groups (*p* > 0.05). Total fiber area in the 0% groups was significantly larger than that in the 12% group (*p* < 0.05), but no significant differences were observed between these groups and the others (*p* > 0.05).

### 3.16. Effects of Different Levels of SPTM on Leg Muscle Fiber Characteristics of Wenchang Chickens

The effects of different levels of SPTM on leg muscle fiber characteristics of Wenchang chickens are presented in Table 17 and Figure 1. As shown in Table 17 and Figure 1, the average fiber area in the 12% group was significantly larger than that in the 0% (*p* < 0.05), but no significant difference was observed between the 3% group and the 9% group (*p* > 0.05). Fiber diameter in the 12% group was significantly larger than that in the 0% and 3% groups (*p* < 0.05).

## 4. Discussion

Although no statistically significant differences were observed in growth performance among treatments, a slight numerical decrease in ADG and an increase in ADFI were observed with increasing dietary SPTM inclusion. One possible explanation is that sweet potato tuber meal contains more structural carbohydrates than conventional corn-based ingredients, which may slightly reduce nutrient utilization efficiency and stimulate compensatory feed intake [18,19]. In addition, because the experiment was conducted during the late fattening phase of Wenchang chickens (81–120 d), a greater proportion of dietary energy may have been directed toward lipid deposition rather than body weight gain. This interpretation is supported by the increased intramuscular fat content observed in the higher SPTM groups. Nevertheless, because these differences were not statistically significant, they should be interpreted with caution.

Meat quality is a key indicator for evaluating poultry production efficiency and consumer acceptance [20]. The formation of meat quality traits is a complex biological process regulated by multiple factors, including muscle moisture content, IMF deposition, amino acid composition, fatty acid profile, and muscle fiber characteristics [21]. Muscle moisture and crude fat contents are major determinants of meat juiciness, tenderness, and sensory acceptability. IMF plays a central role in improving palatability by enhancing flavor release, lubrication during chewing, and overall eating quality [22]. Previous studies demonstrated that moderate increases in IMF significantly improve meat tenderness, juiciness, and consumer preference in poultry and livestock species [23]. Lipids also serve as essential precursors for aroma compounds [24]. During cooking, lipid oxidation and thermal degradation generate volatile aldehydes, ketones, and alcohols that contribute to characteristic meat flavor [25]. Therefore, IMF content is widely used as a critical indicator of meat quality evaluation [26]. In the present study, breast muscle IMF content in the 12% group was significantly higher than that in the control and 3% groups (*p* < 0.05), but no significant difference was observed between the 12% group and the 9% group (*p* > 0.05). These findings suggest that a relatively high inclusion level of SPTM may promote lipid deposition within muscle tissue. Increased IMF deposition has been associated with enhanced sensory properties and improved flavor intensity in indigenous chicken breeds, largely because intramuscular lipids serve as important precursors for volatile flavor compounds generated during cooking [14,27]. However, because physicochemical traits such as pH, color, drip loss, and cooking loss were not significantly affected in the present study, the observed increase in IMF should be interpreted as a selective change in muscle composition rather than as evidence of a broad improvement in overall meat quality. Further studies incorporating sensory evaluation and lipid metabolism analyses are required to clarify the mechanisms underlying the increased IMF deposition induced by dietary SPTM.

IMP is a key flavor nucleotide responsible for umami taste in meat products and is widely recognized as a major determinant of meat palatability [28]. IMP enhances umami perception through synergistic interactions with glutamate and amino acids [29,30]. Accumulation of IMP in muscle is closely associated with postmortem ATP degradation pathways and muscle energy metabolism [31]. In the present study, the 9% sweet potato meal group exhibited significantly higher breast muscle IMP content compared with the control and 3% groups, indicating enhanced umami characteristics. The higher IMP concentration observed in birds fed 9% SPTM may be related to changes in muscle energy metabolism associated with the high content of readily digestible carbohydrates in sweet potato. Previous studies have demonstrated that IMP is generated through the sequential degradation of ATP after slaughter and is an important contributor to the umami taste of chicken meat [27,32]. Nevertheless, because ATP degradation products and energy metabolism-related parameters were not evaluated in the present study, the underlying mechanism remains speculative and warrants further investigation. Comparable improvements in IMP concentration have been reported when dietary energy sources were optimized or alternative carbohydrate ingredients were included in broiler diets [33,34].

Muscle amino acid composition determines both protein nutritional value and flavor properties of chicken meat [35,36]. Different amino acids contribute distinct taste attributes: alanine, glycine, and serine are associated with sweetness, whereas glutamic acid and aspartic acid contribute to umami flavor [37,38]. Sulfur-containing amino acids such as methionine act as important precursors for volatile flavor compounds generated during cooking [39,40]. The present study showed that dietary inclusion of sweet potato meal at 9% did not significantly affect amino acid composition in breast muscle, indicating that partial replacement of conventional energy ingredients did not impair protein nutritional value. Interestingly, dietary supplementation with 12% SPTM resulted in different responses between muscle types. While several amino acid indices were increased in leg muscle, breast muscle exhibited reduced concentrations of total amino acids, essential amino acids, umami amino acids, and several individual amino acids, including Asp, Glu, Thr, and Ser. These findings suggest that the effects of SPTM on amino acid deposition may be muscle-specific rather than uniformly beneficial. Similar tissue-dependent responses have been reported in poultry, where differences in muscle fiber composition, metabolic characteristics, and protein turnover rates between breast and leg muscles resulted in distinct nutrient deposition patterns [41,42,43]. Therefore, the increased amino acid content observed in leg muscle should be interpreted together with the reduced amino acid concentrations detected in breast muscle. The underlying mechanisms remain unclear and warrant further investigation, particularly regarding nutrient partitioning and muscle-specific amino acid metabolism under dietary SPTM supplementation.

Fatty acid composition plays a crucial role in determining meat flavor, texture, juiciness, and oxidative stability [44,45]. Fatty acids such as palmitic acid (C16:0), stearic acid (C18:0), and oleic acid (C18:1) represent major components of muscle lipids and strongly influence sensory quality [46]. Generally, polyunsaturated fatty acids (PUFAs) are more susceptible to oxidation, potentially reducing flavor stability, whereas saturated fatty acids (SFAs) and monounsaturated fatty acids (MUFAs) contribute positively to aroma intensity and tenderness [47,48,49]. The present study demonstrated that dietary supplementation with 12% sweet potato meal increased several fatty acids in both breast and leg muscles. These results indicate that higher inclusion levels modify lipid deposition patterns, likely through enhanced carbohydrate-driven lipogenesis and regulation of lipid metabolism enzymes. Previous studies have similarly shown that dietary carbohydrate level influences fatty acid synthesis and muscle lipid composition in poultry [50].

Muscle fiber characteristics constitute the structural basis of meat tenderness [51,52]. Smaller muscle fiber diameter and higher fiber density are generally associated with reduced shear force and improved tenderness [53,54]. Larger muscle fibers increase myofibrillar packing density, strengthening structural resistance and decreasing tenderness [55,56]. Nutritional regulation has been shown to influence muscle fiber growth patterns and muscle development in poultry [57,58]. In the present study, supplementation with 12% sweet potato meal significantly reduced muscle fiber diameter in breast muscles without affecting fiber density. These findings suggest that sweet potato meal may alter muscle growth dynamics and improve structural characteristics associated with meat tenderness.

## 5. Conclusions

In conclusion, dietary SPTM up to 12% did not negatively affect growth or slaughter performance of Wenchang chickens. Supplementation with 9% SPTM increased breast IMP content, while 12% SPTM increased breast IMF but reduced several amino acid indices. These selective effects on meat quality traits suggest that SPTM has potential as a partial corn replacer, but further studies are needed to optimize inclusion levels and validate sensory outcomes.

## Figures and Tables

**Table 1:** Composition and nutrient levels of diets (air-dry basis,%).

Item	Groups			
	0%	3%	9%	12%
Corn	69.57	65.63	57.90	54.00
SPTM (%):	0.00	3.00	9.00	12.00
Soybean meal	16.95	17.46	18.47	18.99
Fish meal	2.00	2.00	2.00	2.00
Bran	5.00	5.00	5.00	5.00
Soybean oil	3.00	3.43	4.20	4.60
Stone powder	0.96	0.95	0.87	0.85
Calcium hydrogen phosphate	0.93	0.95	0.99	1.00
Salt	0.30	0.30	0.30	0.30
L-lysine salt	1.00	0.09	0.08	0.06
DL- methionine	0.09	0.09	0.09	0.10
Choline chloride	0.10	0.10	0.10	0.10
Pre-mixed powder ^(1)^	1.00	1.00	1.00	1.00
Nutritional Level ^(2)^				
Metabolic energy (MJ/kg)	12.74	12.74	12.73	12.73
Crude protein	15.83	15.83	15.83	15.83
Crude fat	6.13	6.45	6.99	7.27
Calcium	0.79	0.79	0.79	0.79
Total phosphorus	0.61	0.61	0.60	0.60
Effective phosphorus	0.36	0.36	0.37	0.37
Lysine	0.85	0.85	0.85	0.85
Methionine	0.36	0.36	0.36	0.36
Methionine + Cystine	0.64	0.64	0.63	0.63

^(1)^ The premix provided the following per kilogram of diet: vitamin A, 10,000 IU; vitamin D_3_, 2000 IU; vitamin E, 10 mg; vitamin B_1_, 2 mg; vitamin B_2_, 3 mg; vitamin B_6_, 3.50 mg; niacin, 15 mg; copper (Cu), 10 mg; iron (Fe), 80 mg; manganese (Mn), 60 mg; zinc (Zn), 70 mg; iodine (I), 2 mg; selenium (Se), 0.40 mg; folic acid, 0.50 mg; pantothenic acid, 10 mg; biotin, 0.15 mg; and cyanocobalamin (vitamin B_12_), 10 μg. ^(2)^ The calculated values are based on the nutritional value data of raw materials referenced from the “China Feed Composition and Nutritional Value Table (2022)”.

**Table 2:** Effects of different levels of SPTM on growth performance of Wenchang chickens (g).

Item	Initial Body Weight	Final Body Weight	ADG	ADFI	F/G
0% group	1190.45 ± 4.37	1920.93 ± 18.62	18.25 ± 0.33	86.56 ± 1.19	4.74 ± 0.15
3% group	1193.18 ± 6.44	1912.11 ± 38.67	17.98 ± 0.71	86.49 ± 1.60	4.81 ± 0.22
9% group	1188.64 ± 6.63	1893.87 ± 14.48	17.63 ± 0.34	87.36 ± 1.43	4.95 ± 0.17
12% group	1190.91 ± 5.33	1900.45 ± 34.59	17.74 ± 0.65	87.54 ± 1.19	4.93 ± 0.35

**Table 3:** Carcass characteristics of Wenchang chickens fed diets containing different levels of SPTM (%).

Item	Dressing Percentage	Semi-Eviscerated Percentage	Eviscerated Percentage	Abdominal Fat Percentage	Leg Muscle Percentage	Breast Muscle Percentage
0% group	91.05 ± 1.16	68.47 ± 4.21	59.03 ± 3.12	8.11 ± 2.74	20.75± 2.28	16.58 ± 1.92
3% group	91.45 ± 1.63	69.01 ± 2.28	59.01 ± 2.61	8.66 ± 1.33	19.16± 1.57	14.73 ± 1.98
9% group	92.36 ± 1.72	69.28± 4.35	59.86 ± 2.91	8.68 ± 2.45	19.14± 1.89	15.89 ± 3.12
12% group	92.36 ± 1.35	70.24 ± 3.13	59.03 ± 3.12	9.53 ± 1.92	20.62± 2.10	16.40 ± 0.94

**Table 4:** Effects of dietary SPTM supplementation on relative organ weights of Wenchang chickens (g/kg).

Item	Heart Index	Liver Index	Stomach Index	Gizzard Index	Proventriculus Index	Spleen Index
0% group	3.45 ± 0.50	21.24 ± 4.75	12.30 ± 2.03	9.77 ± 1.90	2.53 ± 0.53	2.27 ± 1.41
3% group	3.26 ± 0.81	22.84 ± 7.50	12.87 ± 1.46	10.39 ± 1.34	2.50 ± 0.52	2.47 ± 1.02
9% group	3.22 ± 0.65	22.48 ± 3.77	12.54 ± 1.67	10.27 ± 1.26	2.27 ± 0.58	2.77 ± 1.22
12% group	3.78 ± 1.49	23.28 ± 6.97	12.11 ± 3.36	10.00 ± 2.82	2.11 ± 0.78	3.02 ± 0.88

**Table 5:** Effects of dietary SPTM supplementation on serum biochemical indices of Wenchang chickens.

Item	TP (g/L)	ALB (g/L)	GLB (g/L)	GLU (mmol/L)	UA (μmol/L)	TG (mmol/L)	TC (mmol/L)
0% group	54.530 ± 12.18	17.87 ± 2.01	36.66 ± 10.62	9.93 ± 1.06	223.50 ± 70.50	9.54 ± 4.73	3.97 ± 2.41
3% group	50.89 ± 7.30	16.56 ± 1.23	34.33 ± 7.39	10.07 ± 0.99	183.60 ± 82.13	11.81 ± 7.09	3.52 ± 1.60
9% group	54.53 ± 8.79	17.43 ± 1.18	37.10 ± 8.65	10.11 ± 2.27	284.60 ± 132.23	9.86 ± 7.15	3.80 ± 1.23
12% group	54.02 ± 9.53	18.16 ± 2.91	35.86 ± 6.87	10.56 ± 4.01	249.20 ± 151.81	7.44 ± 7.39	3.70 ± 1.81

**Table 6:** Effects of dietary SPTM supplementation on serum enzyme activities and immune indices of Wenchang chickens.

Item	ALP (U/L)	ALT (U/L)	AST (U/L)	IgA (g/L)	IgM (g/L)
0% group	875.30 ± 646.96	1.30 ± 0.95 ^a^	286.10 ± 60.02 ^ab^	0.01 ± 0.005	0.07 ± 0.01
3% group	617.00 ± 498.40	1.10 ± 0.74 ^a^	249.50 ± 40.09 ^b^	0.04 ± 0.09	0.10 ± 0.14
9% group	971.60 ± 1103.72	0.40 ± 0.52 ^b^	277.10 ± 51.07 ^ab^	0.01 ± 0.02	0.07 ± 0.04
12% group	599.60 ± 298.24	1.10 ± 0.31 ^a^	408.50 ± 202.29 ^a^	0.04 ± 0.08	0.10 ± 0.13

Note: a, b, ab: Different superscript letters within the same column indicate significant differences among treatment groups (*p* < 0.05).

**Table 7:** Effects of dietary SPTM supplementation on jejunal morphological parameters of Wenchang chickens.

Item	Villus Height (mm)	Crypt Depth (mm)	Muscularis Thickness (mm)	Number of Intestinal Glands	Intestinal Gland Area (mm^2^)	Number of Intestinal Glands Per Unit Area (n/mm^2^)
0% group	1.25 ± 0.22 ^a^	0.14 ± 0.02	0.24 ± 0.05	31.93 ± 6.53 ^a^	0.29 ± 0.06	111.84 ± 16.18 ^a^
3% group	1.05 ± 0.30 ^ab^	0.16 ± 0.03	0.25 ± 0.05	25.83 ± 5.95 ^ab^	0.28 ± 0.05	95.00 ± 22.26 ^ab^
9% group	0.91 ± 0.28 ^b^	0.16 ± 0.02	0.27 ± 0.04	24.47 ± 6.31 ^b^	0.28 ± 0.06	87.60 ± 11.79 ^b^
12% group	1.24 ± 0.36 ^a^	0.15 ± 0.04	0.24 ± 0.04	27.30 ± 8.43 ^ab^	0.25 ± 0.03	110.91 ± 37.10 ^a^

Note: a, b, ab: Different superscript letters within the same column indicate significant differences among treatment groups (*p* < 0.05).

**Table 8:** Effects of dietary SPTM supplementation on physicochemical properties of breast muscle in Wenchang chickens.

Item	pH_45min_	pH_24h_	Drip Loss (%)	L*	a*	b*
0% group	5.70 ± 0.17	5.67 ± 0.44	3.92 ± 1.67	47.27 ± 2.58	−0.26 ± 1.64	8.36 ± 1.23
3% group	5.69 ± 0.35	5.82 ± 0.25	4.47 ± 2.63	47.96 ± 1.02	−1.29 ± 0.31	5.36 ± 0.94
9% group	5.66 ± 0.16	6.03 ± 0.33	3.39 ± 0.21	48.65 ± 3.94	−0.78 ± 0.49	5.31 ± 3.07
12% group	5.65 ± 0.20	5.82 ± 0.29	3.03 ± 1.03	48.03 ± 4.62	−0.39 ± 0.79	7.78 ± 1.89

Note: lightness (L*), redness (a*), or yellowness (b*). “−” indicates a negative value.

**Table 9:** Effects of dietary SPTM supplementation on physicochemical properties of leg muscle in Wenchang chickens.

Item	pH_45min_	pH_24h_	Drip Loss (%)	L*	a*	b*
0% group	5.81 ± 0.10	6.00 ± 0.39	2.85 ± 0.73	50.87 ± 3.17	1.29 ± 1.93	12.74 ± 3.24
3% group	5.90 ± 0.23	6.00 ± 0.22	3.58 ± 1.39	51.59 ± 2.61	0.63 ± 1.70	10.24 ± 3.45
9% group	5.89 ± 0.08	6.16 ± 0.23	3.91 ± 1.13	52.28 ± 4.95	0.32 ± 0.54	10.50 ± 1.26
12% group	5.87 ± 0.13	6.18 ± 0.18	3.33 ± 0.67	50.29 ± 2.18	2.33 ± 0.50	11.63 ± 1.95

Note: lightness (L*), redness (a*), or yellowness (b*).

**Table 10:** Effects of dietary SPTM supplementation on nutrient composition of breast muscle in Wenchang chickens (g/100 g).

Item	Moisture	Crude Protein	IMF	IMP
0% group	73.15 ± 2.23 ^a^	23.78 ± 2.10	1.05 ± 0.41 ^b^	1.12 ± 0.31 ^b^
3% group	70.65 ± 1.48 ^ab^	23.28 ± 0.98	1.43 ± 0.59 ^b^	1.16 ± 0.18 ^b^
9% group	72.35 ± 0.48 ^a^	22.65 ± 1.00	1.75 ± 1.42 ^ab^	1.75 ± 0.44 ^a^
12% group	68.50 ± 1.66 ^b^	22.58 ± 1.48	4.30 ± 3.61 ^a^	1.42 ± 0.40 ^ab^

Note: a, b, ab: Different superscript letters within the same column indicate significant differences among treatment groups (*p* < 0.05).

**Table 11:** Effects of dietary SPTM supplementation on nutrient composition of leg muscle in Wenchang chickens (g/100 g).

Item	Moisture	Crude Protein	IMF	IMP
0% group	67.88 ± 4.15	19.30 ± 1.47	8.30 ± 4.66	0.61 ± 0.13
3% group	67.90 ± 1.24	18.98 ± 1.05	9.43 ± 2.39	0.74 ± 0.09
9% group	67.55 ± 2.33	18.35 ± 1.31	6.93 ± 3.45	0.70 ± 0.14
12% group	66.73 ± 3.88	19.38 ± 3.46	8.45 ± 5.96	0.83 ± 0.36

**Table 12:** Effects of dietary SPTM supplementation on amino acid composition of breast muscle in Wenchang chickens (g/100 g).

Item	0% Group	3% Group	9% Group	12% Group
Total amino acids (TAAs)	21.53 ± 0.90 ^a^	21.03 ± 0.83 ^a^	20.40 ± 0.79 ^ab^	18.88 ± 1.74 ^b^
Essential amino acids (EAAs)	9.14 ± 0.44 ^a^	8.95 ± 0.34 ^a^	8.68 ± 0.30 ^a^	7.98 ± 0.79 ^b^
Umami amino acids (UAAs)	9.56 ± 0.32 ^a^	9.31 ± 0.30 ^a^	9.04 ± 0.35 ^a^	8.40 ± 0.69 ^b^
Aspartic acid (Asp)	2.21 ± 0.07 ^a^	2.19 ± 0.08 ^a^	2.07 ± 0.07 ^a^	1.91 ± 0.19 ^b^
Threonine (Thr)	1.04 ± 0.03 ^a^	1.04 ± 0.04 ^a^	0.98 ± 0.04 ^ab^	0.92 ± 0.07 ^b^
Serine (Ser)	0.88 ± 0.02 ^a^	0.87 ± 0.04 ^a^	0.82 ± 0.03 ^b^	0.79 ± 0.04 ^b^
Glutamic acid (Glu)	3.45 ± 0.13 ^a^	3.37 ± 0.11 ^a^	3.26 ± 0.16 ^a^	3.05 ± 0.18 ^b^
Proline (Pro)	0.54 ± 0.02	0.57 ± 0.06	0.57 ± 0.02	0.56 ± 0.04
Glycine (Gly)	0.74 ± 0.02	0.80 ± 0.06	0.79 ± 0.03	0.80 ± 0.08
Alanine (Ala)	1.01 ± 0.03	1.06 ± 0.06	1.08 ± 0.05	1.11 ± 0.13
Valine (Val)	0.81 ± 0.03	0.90 ± 0.08	0.84 ± 0.04	0.89 ± 0.12
Methionine (Met)	0.43 ± 0.05 ^b^	0.45 ± 0.05 ^ab^	0.50 ± 0.03 ^a^	0.51 ± 0.06 ^a^
Isoleucine (Ile)	0.85 ± 0.03	0.94 ± 0.07	0.91 ± 0.04	0.93 ± 0.10
Leucine (Leu)	1.38 ± 0.05 ^b^	1.59 ± 0.12 ^a^	1.53 ± 0.09 ^ab^	1.56 ± 0.17 ^a^
Tyrosine (Tyr)	0.70 ± 0.03 ^b^	0.78 ± 0.04 ^a^	0.74 ± 0.03 ^ab^	0.71 ± 0.03 ^b^
Phenylalanine (Phe)	0.71 ± 0.02 ^b^	0.77 ± 0.04 ^ab^	0.79 ± 0.04 ^a^	0.78 ± 0.07 ^a^
Lysine (Lys)	1.70 ± 0.06 ^b^	1.91 ± 0.13 ^a^	1.83 ± 0.10 ^ab^	1.88 ± 0.17 ^ab^
Histidine (His)	0.46 ± 0.03	0.51 ± 0.06	0.47 ± 0.03	0.60 ± 0.23
Arginine (Arg)	1.15 ± 0.03	1.23 ± 0.10	1.16 ± 0.06	1.22 ± 0.12

Note: a, b, ab: Different superscript letters within the same row indicate significant differences among treatment groups (*p* < 0.05).

**Table 13:** Effects of dietary SPTM supplementation on amino acid composition of leg muscle in Wenchang chickens (g/100 g).

Item	0% Group	3% Group	9% Group	12% Group
Total amino acids (TAAs)	16.30 ± 0.42 ^b^	18.13 ± 1.26 ^a^	17.53 ± 0.91 ^ab^	18.00 ± 1.77 ^ab^
Essential amino acids (EAAs)	6.65 ± 0.18 ^b^	7.44 ± 0.52 ^a^	7.23 ± 0.38 ^ab^	7.42 ± 0.78 ^a^
Umami amino acids (UAAs)	7.51 ± 0.20 ^b^	8.32 ± 0.53 ^a^	8.12 ± 0.41 ^ab^	8.21 ± 0.64 ^a^
Aspartic acid (Asp)	1.59 ± 0.05 ^b^	1.82 ± 0.14 ^a^	1.73 ± 0.10 ^ab^	1.78 ± 0.18 ^a^
Threonine (Thr)	0.76 ± 0.03 ^b^	0.89 ± 0.06 ^a^	0.84 ± 0.05 ^ab^	0.87 ± 0.09 ^a^
Serine (Ser)	0.72 ± 0.03 ^b^	0.82 ± 0.07 ^a^	0.77 ± 0.05 ^ab^	0.78 ± 0.07 ^ab^
Glutamic acid (Glu)	2.75 ± 0.08 ^b^	3.09 ± 0.20 ^a^	3.01 ± 0.17 ^a^	3.04 ± 0.20 ^a^
Proline (Pro)	0.49 ± 0.03	0.51 ± 0.04	0.52 ± 0.03	0.51 ± 0.02
Glycine (Gly)	0.78 ± 0.03	0.82 ± 0.04	0.79 ± 0.02	0.81 ± 0.05
Alanine (Ala)	1.02 ± 0.04	1.03 ± 0.02	1.05 ± 0.06	1.13 ± 0.03
Valine (Val)	0.82 ± 0.05	0.91 ± 0.05	0.82 ± 0.01	0.85 ± 0.02
Methionine (Met)	0.41 ± 0.01 ^a^	0.52 ± 0.03 ^ab^	0.52 ± 0.05 ^b^	0.54 ± 0.03 ^b^
Isoleucine (Ile)	0.81 ± 0.02	0.92 ± 0.11	0.93 ± 0.03	0.92 ± 0.11
Leucine (Leu)	1.41 ± 0.02^a^	1.58 ± 0.02 ^b^	1.55 ± 0.07 ^ab^	1.58 ± 0.13 ^b^
Tyrosine (Tyr)	0.68 ± 0.06 ^a^	0.75 ± 0.03 ^b^	0.78 ± 0.06 ^b^	0.70 ± 0.04 ^ab^
Phenylalanine (Phe)	0.72 ± 0.03	0.73 ± 0.05	0.78 ± 0.03	0.74 ± 0.08
Lysine (Lys)	1.63 ± 0.03 ^a^	1.88 ± 0.08 ^ab^	1.86 ± 0.06 ^ab^	1.83 ± 0.07 ^ab^
Histidine (His)	0.43 ± 0.04	0.52 ± 0.03	0.48 ± 0.01	0.62 ± 0.10
Arginine (Arg)	1.12 ± 0.06	1.13 ± 0.11	1.13 ± 0.06	1.20 ± 0.10

Note: a, b, ab: Different superscript letters within the same row indicate significant differences among treatment groups (*p* < 0.05).

**Table 14:** Effects of dietary SPTM supplementation on the fatty acid profile of breast muscle in Wenchang chickens (g/100 g).

Item	0% Group	3% Group	9% Group	12% Group
Total fatty acids (TFAs)	2.27 ± 1.04 ^b^	3.84 ± 1.34 ^ab^	2.69 ± 0.63 ^b^	5.72 ± 1.76 ^a^
Saturated fatty acids (SFAs)	0.75 ± 0.34 ^b^	1.22 ± 0.42 ^ab^	0.85 ± 0.18 ^b^	1.84 ± 0.52 ^a^
Monounsaturated fatty acids (MUFAs)	0.12 ± 0.06 ^b^	0.18 ± 0.05 ^ab^	0.12 ± 0.03 ^b^	0.31 ± 0.15 ^a^
Polyunsaturated fatty acids (PUFAs)	0.58 ± 0.23 ^b^	1.04 ± 0.38 ^ab^	0.70 ± 0.20 ^b^	1.31 ± 0.42 ^a^
Myristic acid (C14:0)	0.02 ± 0.01 ^b^	0.04 ± 0.02 ^b^	0.04 ± 0.01 ^b^	0.10 ± 0.02 ^a^
Palmitic acid (C16:0)	0.55 ± 0.26 ^b^	0.88 ± 0.29 ^ab^	0.61 ± 0.12 ^b^	1.35 ± 0.43 ^a^
cis-9-Hexadecenoic acid (C16:1)	0.07 ± 0.04 ^b^	0.11 ± 0.04 ^b^	0.07 ± 0.02 ^b^	0.20 ± 0.11 ^a^
Stearic acid (C18:0)	0.18 ± 0.08 ^b^	0.30 ± 0.11 ^ab^	0.20 ± 0.05 ^b^	0.38 ± 0.07 ^a^
cis-9-Octadecenoic acid (C18:1)	0.83 ± 0.41 ^b^	1.37 ± 0.49 ^ab^	0.98 ± 0.23 ^b^	2.15 ± 0.68 ^a^
cis,cis-9,12-Octadecadienoic acid (C18:2n-6)	0.49 ± 0.22 ^b^	0.94 ± 0.37 ^ab^	0.61 ± 0.20 ^b^	1.24 ± 0.41 ^a^
cis-11-Eicosenoic acid (C20:1)	0.01 ± 0.01 ^b^	0.01 ± 0.01 ^ab^	0.01 ± 0.00 ^b^	0.02 ± 0.00 ^a^
α-Linolenic acid (C18:3n-3)	0.02 ± 0.01 ^b^	0.04 ± 0.01 ^ab^	0.02 ± 0.01 ^b^	0.05 ± 0.02 ^a^
cis,cis-11,14-Eicosadienoic acid (C20:2)	0.00 ± 0.00 ^b^	0.01 ± 0.00 ^ab^	0.01 ± 0.00 ^b^	0.01 ± 0.00 ^a^
Arachidonic acid (C20:4n-6)	0.07 ± 0.01 ^a^	0.07 ± 0.00 ^a^	0.06 ± 0.01 ^ab^	0.05 ± 0.00 ^b^
Docosahexaenoic acid (DHA, C22:6n-3)	0.01 ± 0.00 ^a^	0.01 ± 0.00 ^ab^	0.01 ± 0.00 ^a^	0.01 ± 0.00 ^b^

Note: a, b, ab: Different superscript letters within the same row indicate significant differences among treatment groups (*p* < 0.05).

**Table 15:** Effects of dietary SPTM supplementation on fatty acid composition of leg muscle in Wenchang chickens (g/100 g).

Item	0% Group	3% Group	9% Group	12% Group
Total fatty acids (TFAs)	10.54 ± 3.15	9.59 ± 1.87	10.09 ± 2.40	8.88 ± 1.93
Saturated fatty acids (SFAs)	3.32 ± 1.11	3.07 ± 0.64	3.23 ± 0.60	2.97 ± 0.65
Monounsaturated fatty acids (MUFAs)	4.66 ± 1.34	3.85 ± 0.77	4.36 ± 1.32	3.79 ± 0.84
Polyunsaturated fatty acids (PUFAs)	2.54 ± 0.70	2.67 ± 0.53	2.51 ± 0.54	2.11 ± 0.51
Lauric acid (C12:0)	0.01 ± 0.01 ^c^	0.05 ± 0.02 ^b^	0.12 ± 0.02 ^a^	0.14 ± 0.05 ^a^
Myristic acid (C14:0)	0.08 ± 0.03 ^b^	0.11 ± 0.03 ^b^	0.16 ± 0.01 ^a^	0.16 ± 0.05 ^a^
cis-9-Tetradecenoic acid (C14:1)	0.01 ± 0.00	0.01 ± 0.00	0.01 ± 0.01	0.02 ± 0.00
Pentadecanoic acid (C15:0)	0.01 ± 0.00	0.01 ± 0.00	0.01 ± 0.00	0.01 ± 0.00
Palmitic acid (C16:0)	2.51 ± 0.81 ^a^	2.17 ± 0.41 ^ab^	2.24 ± 0.52 ^ab^	2.07 ± 0.43 ^ab^
cis-9-Hexadecenoic acid (C16:1)	0.45 ± 0.11	0.31 ± 0.05	0.36 ± 0.18	0.34 ± 0.10
Heptadecanoic acid (C17:0)	0.02 ± 0.00	0.02 ± 0.01	0.02 ± 0.00	0.01 ± 0.00
Stearic acid (C18:0)	0.69 ± 0.25	0.69 ± 0.17	0.66 ± 0.08	0.58 ± 0.14
trans-9-Octadecenoic acid (C18:1t)	0.02 ± 0.00	0.01 ± 0.00	0.01 ± 0.00	0.01 ± 0.00
cis-9-Octadecenoic acid (C18:1)	4.08 ± 1.19	3.42 ± 0.72	3.87 ± 1.11	3.34 ± 0.74
cis,cis-9,12-Octadecadienoic acid (C18:2n-6)	2.38 ± 0.67	2.48 ± 0.52	2.34 ± 0.53	1.95 ± 0.51
Eicosanoic acid (C20:0)	0.01 ± 0.00	0.01 ± 0.00	0.01 ± 0.00	0.01 ± 0.00
cis,cis,cis-6,9,12-Octadecatrienoic acid (C18:3n-6)	0.02 ± 0.01	0.01 ± 0.00	0.01 ± 0.00	0.02 ± 0.01
cis-11-Eicosenoic acid (C20:1)	0.04 ± 0.01	0.04 ± 0.01	0.04 ± 0.01	0.04 ± 0.01
α-Linolenic acid (C18:3n-3)	0.10 ± 0.03	0.10 ± 0.02	0.10 ± 0.03	0.08 ± 0.02
cis,cis-11,14-Eicosadienoic acid (C20:2)	0.02 ± 0.01	0.02 ± 0.01	0.02 ± 0.00	0.02 ± 0.00
cis,cis,cis-8,11,14-Eicosatrienoic acid (C20:3n-6)	0.01 ± 0.01	0.01 ± 0.00	0.01 ± 0.00	0.01 ± 0.00
Arachidonic acid (C20:4n-6)	0.07 ± 0.01 ^b^	0.09 ± 0.01 ^a^	0.07 ± 0.01 ^b^	0.07 ± 0.01 ^b^
Docosahexaenoic acid (DHA, C22:6n-3)	0.01 ± 0.00	0.01 ± 0.00	0.01 ± 0.00	0.01 ± 0.00

Note: a, b, c, ab: Different superscript letters within the same row indicate significant differences among treatment groups (*p* < 0.05).

**Table 16:** Effects of dietary SPTM supplementation on breast muscle fiber characteristics in Wenchang chickens.

Item	Number of Fibers	Total Fiber Area (mm^2^)	Average Fiber Area (µm^2^)	Fiber Density (n/mm^2^)	Fiber Diameter (µm)
0% group	51.87 ± 12.70 ^a^	0.10 ± 0.01 ^a^	1.70 ± 0.60 ^b^	685.29 ± 221.99	48.00 ± 9.00 ^b^
3% group	43.30 ± 13.07 ^ab^	0.09 ± 0.00 ^ab^	1.80 ± 0.40 ^b^	587.21 ± 126.29	50.00 ± 5.00 ^b^
9% group	41.33 ± 9.38 ^ab^	0.09 ± 0.01 ^ab^	2.00 ± 0.60 ^ab^	596.43 ± 174.45	51.00 ± 8.00 ^ab^
12% group	40.03 ± 9.39 ^b^	0.08 ± 0.01 ^b^	2.60 ± 1.40 ^a^	558.07 ± 167.52	53.00 ± 7.00 ^ab^

Note: a, b, ab: Different superscript letters within the same column indicate significant differences among treatment groups (*p* < 0.05).

**Table 17:** Effects of dietary SPTM supplementation on leg muscle fiber characteristics in Wenchang chickens.

Item	Number of Fibers	Total Fiber Area (mm^2^)	Average Fiber Area (µm^2^)	Fiber Density (n/mm^2^)	Fiber Diameter (µm)
0% group	58.33 ± 21.10	0.09 ± 0.01 ^a^	1.80 ± 0.40 ^b^	572.54 ± 147.34 ^a^	56.00 ± 6.00 ^a^
3% group	50.23 ± 13.75	0.09 ± 0.01 ^ab^	2.20 ± 0.70 ^ab^	492.96 ± 138.80 ^ab^	60.00 ± 8.00 ^b^
9% group	48.27 ± 14.52	0.09 ± 0.01 ^ab^	2.20 ± 0.40 ^ab^	475.65 ± 104.88 ^ab^	60.00 ± 4.00 ^b^
12% group	41.77 ± 23.38	0.08 ± 0.01 ^b^	2.20 ± 0.50 ^ab^	490.78 ± 132.61 ^ab^	58.00 ± 4.00 ^b^

Note: a, b, ab: Different superscript letters within the same column indicate significant differences among treatment groups (*p* < 0.05).

## Data Availability

The original contributions generated for this study are included in the article. Further inquiries can be directed to the corresponding author.
